# Identify MTDH as a Key Gene of Radio-Resistance in Colorectal Cancer Based on Multi-Omics and Experimental Validation

**DOI:** 10.32604/or.2026.075314

**Published:** 2026-04-22

**Authors:** Wei Xu, Yuanyuan Zhang, Yizhi Ge, Yesong Guo

**Affiliations:** 1 Department of Oncology, Suqian Hospital of Traditional Chinese Medicine, Suqian, China; 2 Department of Radiation Oncology, Jiangsu Cancer Hospital & Jiangsu Institute of Cancer Research & The Affiliated Cancer Hospital of Nanjing Medical University, Nanjing, China

**Keywords:** Colorectal cancer, MTDH, radio-resistance, multi-omics, experiment

## Abstract

**Objectives:**

Radio-resistance hinders the effectiveness of radiotherapy for treating colorectal cancer (CRC) patients. Metadherin (MTDH) is proposed to exert a pivotal role in resistance to radiotherapy in various malignancies. This study aims to investigate the precise impact of MTDH on CRC radio-resistance.

**Methods:**

Through a fusion of 14 machine learning algorithms and SHapley Additive exPlanations (SHAP) interpretability analysis, we pinpointed MTDH as a pivotal gene implicated in radio-resistance mechanisms. Subsequently, we investigated MTDH expression in CRC tissues using single-cell RNA sequencing data (scRNA-seq) and bulk transcriptomic data. MTDH level was also examined in tissues from 82 rectal cancer patients who were responsive or non-responsive to radiotherapy. We established radioresistant variants of SW480 and HT29 cells (designated SW480-R and HT29-R), then evaluated their characteristics using cell viability assays, apoptosis measurements, and γ-H2AX foci immunofluorescence. Then, MTDH silencing in radioresistant cells was applied to further investigate the impact of MTDH on regulating radiosensitivity for CRC cells.

**Results:**

Machine learning analysis revealed a significant association between MTDH and radio-resistance. Furthermore, multi-omics data confirmed that MTDH expression was significantly upregulated in CRC tissues and, more notably, within the more malignant diploid single-cell subpopulation. Genes associated with MTDH were predominantly enriched in pathways related to damage repair, DNA damage response, epithelial-mesenchymal transition (EMT), and stem cell differentiation, which were known to be critically involved in radio-resistance. Experimental validation confirmed significantly elevated MTDH expression in both radioresistant rectal cancer specimens and corresponding cellular models. High level of MTDH was positively related to several clinicopathological parameters, including tumor stage, differentiation, and lymph node status. Silencing of MTDH inhibited proliferative ability, increased apoptosis, and increased γ-H2AX foci numbers in CRC cells with radiation treatment.

**Conclusion:**

This study emphasizes the potential of MTDH as a promising prognostic and therapeutic target in response to radiotherapy for treating CRC patients.

## 1 Introduction

Colorectal cancer (CRC) is a prevalent malignancy occurring in the intestinal epithelium that is frequently diagnosed at advanced stages due to its high potential for distant metastasis [1–3]. Furthermore, CRC imposes substantial incidence and mortality internationally, with over 1.85 million cases and as many as 850,000 deaths each year [3]. Currently, radiotherapy is deemed one of the most common antitumor therapeutic regimens. Radiotherapy is often administered as adjuvant therapy after initial surgical treatment. Radiation therapy can also be used with curative or palliative intent and as neoadjuvant therapy prior to surgical treatment [4]. Radiotherapy has been extensively used to treat rectal cancer [5]. Both preoperative and postoperative radiation therapy significantly lowers the risk of local recurrence and prolongs the survival time of rectal cancer patients [6]. However, despite the importance of radiotherapy for locally advanced rectal cancer, there is still less than one-fifth of patients achieve a pathological complete response, which indicates that radiation resistance has emerged as one of the primary factors limiting the effectiveness of this treatment [7]. Resistance to radiation can increase the risk of local recurrence and distal metastasis [8]. Therefore, a deeper understanding of the mechanisms underlying radio-resistance and seeking possible targets is desperately essential for developing more effective treatments against CRC.

Metadherin (MTDH), a transmembrane protein consisting of 582-amino [9]. MTDH is able to modulate various biological processes, which makes it a potential critical target for the initiation and development in multiple types of human malignancies [10,11]. For instance, MTDH is overexpressed in glioma cells and can mediate oncogenicity via augmenting apoptosis and promoting cell proliferation [12]. Silencing of MTDH leads to accelerated apoptosis in pancreatic cancer cells [13]. Furthermore, similar results are obtained that MTDH is involved in the context of lung cancer and CRC. Importantly, MTDH also serves a pivotal role in treatment resistance [14]. Depletion of MTDH heightens sensitivity to cisplatin in endometrial cancer cells, accompanied by impaired cell viability [15]. The protein expression of MTDH was found to be elevated in radiotherapy-resistant triple-negative breast cancer cells [16]. However, the exact impact of MTDH on CRC radiation resistance remains unclear.

In the present study, we aimed to clarify the correlation between MTDH expression and radiosensitivity in CRC, which could help provide new insights into the critical mediator of MTDH for the radiosensitivity of CRC.

## 2 Material and Methods

### 2.1 Collection of Gene Expression Omnibus (GEO) Datasets

We obtained CRC transcriptome and single-cell RNA sequencing data (scRNA-seq) from GEO (http://www.ncbi.nlm.nih.gov/geo/). The analysis included bulk RNA-seq cohorts of patients undergoing preoperative radiotherapy bulk RNA-seq cohorts (GSE35452, GSE68204, GSE145037, GSE150082) and one raw scRNA-seq dataset (GSE166555). GSE35452 contains 46 cases, including 24 preoperative radiotherapy rectum adenocarcinoma (READ) responders and 22 preoperative radiotherapy non-responders. GSE68204 contains 59 cases, including 38 and 21 locally advanced rectal cancer (LARC) patients, respectively, for a total of 32 non-responders and 27 responders. GSE145037 contains 31 LARC patients receiving preoperative chemoradiotherapy (CRT). GSE150082 contains 39 cases, including 16 sensitive and 23 resistant READ patients who received standard long-course pelvic radiotherapy and capecitabine chemotherapy. GSE166555 contains 12 tumor tissues and their paracancer controls. For the scRNA-seq dataset, raw count matrices were obtained, and quality control, normalization, and cell-type annotation were performed *de novo* using the Seurat R package (v4.0), independent of the original study’s pipeline.

### 2.2 Screening of Differentially Expressed Genes (DEGs)

The “limma” package in R (version 3.62.1) was employed to analyze differential expression in dataset GSE68204. Prior to differential expression analysis, raw data were background-corrected and normalized using the Robust Multi-array Average (RMA) algorithm, which implements quantile normalization. For comparing radioresistant vs. radiosensitive samples, a design matrix was constructed to compare radioresistant vs. radiosensitive samples, without inclusion of additional covariates, with a threshold of *p* value < 0.05 (Benjamini-Hochberg adjusted *p*-value) and |log_2_Fold Change (FC)| > 1.

### 2.3 Construction and Validation of the Machine Learning-Based Predictive Model

To build diagnostic models for forecasting the effectiveness of pre-radiotherapy treatment, batch effect correction was first performed on the GEO datasets using the ComBat algorithm implemented in the “sva” R package [17]. The originating dataset source was treated as the batch variable. To prevent the removal of biological signals, treatment response status (radioresistant vs. radiosensitive) was explicitly included as a covariate of interest to be preserved during the correction process. 14 machine learning algorithms (GlmBoost, XgBoost, Ranger, Stepglm, RF, NaiveBayes, Ridge, Enet, SVM, K-nearest neighbor (KNN), Lasso, multiNom, LDA, and LogReg) were used in the GSE68204 cohort. To ensure the reproducibility and robustness of the results, a 5-fold cross-validation framework was implemented. To prevent data leakage, hyperparameter optimization was performed via grid search nested within the training folds. To address potential class imbalance within the training set, the Synthetic Minority Over-sampling Technique (SMOTE) was applied exclusively to the training data within each cross-validation fold [18]. The original class distribution in the analyzed GSE68204 cohort was 32 radioresistant vs. 27 radiosensitive samples. After applying SMOTE within the training loops, the class distribution was balanced to approximately 1:1. The “optimal model” was defined as the one achieving the highest average Area Under the Curve (AUC) across the cross-validation folds. To identify pivotal radio-resistance-associated genes, we employed SHapley Additive exPlanations (SHAP) on this best-performing model, a method that provides a quantitative interpretation of machine learning models by assessing feature importance [19]. To ensure a comprehensive and stable interpretation of the model’s decision-making process, SHAP values were computed based on the entire GSE68204 cohort. This approach allows for the quantification of both positive and negative contributions of each radio-resistance-associated gene, thereby enhancing model transparency and clinical interpretability [20]. As the SHAP value approached 0, the possibility of further deleting input variables (radio-resistance-associated genes) increased (the SHAP value was approximately 0), ultimately improving the net benefits of the prediction model. The *Y*-axis refers to various characteristic variables (radio-resistance-associated genes), the *X*-axis refers to variable SHAP values; data jitter (data metastable state) reflects the distribution of SHAP values, and the order of the variables represents their importance [21]. External validation using datasets GSE35452, GSE145037, and GSE150082. To strictly prevent data leakage, batch correction was not performed across the training and validation cohorts; instead, the validation datasets were normalized independently (z-score transformation) to ensure scale consistency. The model trained on the GSE68204 cohort was directly applied to these external datasets without retraining, utilizing the identical set of gene features identified in the discovery phase. Predictive accuracy was assessed by computing Receiver Operating Characteristic (ROC) curves and AUC values using the ‘pROC’ R package, based on the model’s predicted probabilities and the true clinical response labels.

### 2.4 scRNA-Seq Data Processing

The scRNA-seq data of CRC from GSE166555 were processed using the Seurat package (V4.0) to create a Seurat object. Cells were filtered based on the following criteria: genes detected <250 or >5000, and mitochondrial contamination >25%. We employed the “normalizedata”, “findcariablefeatures”, and “scaledata” functions from the Seurat package for data normalization, selection of 3000 highly variable genes, in accordance with current best practices for complex single-cell dataset integration [22]. Principal Component Analysis (PCA) was performed firstly. Subsequently, the ‘RunHarmony’ function was applied to the top 30 principal components (PCs) for data integration, using ‘Patient ID’ as the batch variable. To identify cell clusters, we utilized Seurat’s graph-based clustering approach. First, a KNN graph was constructed using the ‘FindNeighbors’ function based on the top 30 PCs. Subsequently, clusters were identified using the ‘FindClusters’ function with a resolution parameter of 0.5. Finally, we performed dimensionality reduction through Uniform Manifold Approximation and Projection (UMAP) to generate two-dimensional visualizations of the identified cell clusters. Inter-cluster differentially expressed genes were detected using Seurat’s “FindAllMarkers” function with the Wilcoxon-Mann-Whitney test. To control for multiple comparisons, statistically significant markers were identified based on a Bonferroni-adjusted *p*-value < 0.01. Additionally, a stringent screening criterion of |log_2_FC| > 2 was applied to prioritize the most robust and highly discriminatory markers for defining distinct cell identities, minimizing the inclusion of low-confidence or noisy features commonly associated with scRNA-seq data. To distinguish diploid and aneuploid cells, the “copykat” package was used to identify epithelial cells in the single-cell data. The “dplyr” package was then employed to integrate the diploid and aneuploid subpopulation information obtained from copykat into the metadata of the Seurat object.

### 2.5 Expression Analysis of MTDH

MTDH expression (The Cancer Genome Atlas (TCGA) tumor types and corresponding GTEx normal tissues) was analyzed at the transcriptomic level via Gene Expression Profiling Interactive Analysis (GEPIA) 2.0 (TCGA-based) (http://gepia2.cancer-pku.cn/), comparing tumor-normal samples (log_2_FC cutoff > 1, *q*-value < 0.01). To validate the translational relevance of the identified gene signatures at the protein level, protein abundance was assessed using Immunohistochemistry (IHC) data from the Human Protein Atlas (HPA) repository (https://www.proteinatlas.org/). Specifically, the IHC staining patterns served as supportive qualitative evidence to corroborate the transcriptomic findings.

### 2.6 Functional Enrichment Analysis

We employed the Cluster Profiler package in R (version v4.10.0) for gene ID conversion within gene sets, for Kyoto Encyclopedia of Genes and Genomes (KEGG) and Gene Ontology (GO) enrichment analysis were performed using *enrichKEGG* and *enrichGO*, and we applied the barplot and dotplot functions from the clusterProfiler package to generate bar charts and biological signal bubble plots, respectively.

### 2.7 Gene Set Enrichment Analysis (GSEA)

GSEA was performed to search for the significant pathways for MTDH-associated DEGs (all detected genes were included and ranked based on the Signal-to-Noise Ratio (Signal2Noise) between MTDH-high and MTDH-low expression groups). The analysis was conducted using GSEA software (version 4.1.0) with the number of permutations set to 1000. The gene set database used was c5.go.v7.2.symbols.gmt, obtained from the Molecular Signatures Database (MSigDB). Statistically significant enriched gene sets were identified based on a nominal *p*-value < 0.05 and a False Discovery Rate (FDR) *q*-value < 0.25.

### 2.8 Human Participants and Tissue Specimen Collection

This study utilized a cohort of human tissue samples obtained from pre-treatment colonoscopy procedures, comprising matched tumor and adjacent normal tissues from rectal cancer patients undergoing neoadjuvant chemoradiotherapy (nCRT) in Jiangsu Cancer Hospital from September 2022 to September 2024. Samples were stored in liquid nitrogen following collection. The specimens were collected from patients without any anti-tumor therapies, including immunotherapy, targeted therapy, biological therapy, and surgery. Inclusion criteria: (1) Age greater than 18; (2) Patients with T2–4 and/or N+ (node-positive) rectal adenocarcinoma; (3) ECOG performance status of 0, 1, or 2; (4) Tumor distal pole located within 12 cm from the anal verge. Exclusion criteria: Patients with metastatic disease, recurrent cancer, and previous pelvic surgery and radiation. Treatment response to nCRT was evaluated postoperatively according to AJCC TRG criteria (0–3). Based on assessments by two pathologists, patients were stratified into Response (TRG 0–1) and Non-response (TRG 2–3) groups [23]. The research was carried out in compliance with the Declaration of Helsinki (Revised 2013) and received approval from the Ethics Committee of Jiangsu Cancer Hospital (Approval Number: KY-2024-142). All participants were provided with information regarding the study and gave their written informed consent prior to participation.

### 2.9 Cell Culture and Treatment

The normal epithelial cell line NCM460 (CRL-1642) and CRC cell lines (LOVO (CCL-229), HT29 (HTB-38), SW620 (CCL-227), and SW480 (CCL-228)) were procured from American Type Culture Collection (ATCC, Manassas, VA, USA). All the cells were cultivated in RPMI-1640 medium (Gibco, 11875093, Grand Island, NY, USA), comprising 10% fetal bovine serum (FBS, Solarbio, 164210-50, Beijing, China), 100 U/mL penicillin, and 100 μg/mL streptomycin (P/S, P1400, Solarbio) at 37°C and 5% CO_2_.

### 2.10 Establishment of CRC Radioresistant Cells and Irradiation Treatment

Cells (SW480/HT29) were irradiated with 4 Gy fractions (radiation dose rate of 5 Gy/min) via PRIMUS X-ray system (Siemens, PRIMUS, Erlangen, Germany) until accumulating 40 Gy total dose [24]. The established radioresistant sublines (designated SW480-R and HT29-R) were maintained irradiation-free for a minimum of three weeks before experimentation. Cells are passaged every 4 days in a 1:3 ratio. To assess radiosensitivity, cells were exposed to either escalating radiation doses (0, 2, 4, 6 Gy) (single fraction) or a standardized 4 Gy dose. Each dose was performed 3 replicates.

### 2.11 Cell Transfection

MTDH suppressor small interfering (si) siRNAs or si-negative control (NC) synthesized by GenePharma (Shanghai, China) were transfected into SW480-R and HT29-R cells using Lipofectamine 3000 (Invitrogen, L3000015, Carlsbad, CA, USA), with a final siRNA concentration of 60–100 nM. Following validation was conducted through the utilization of quantitative real-time polymerase chain reaction (qRT-PCR) and western blotting. The siRNA sequences used were: si-NC: TTCTCCGAACGTGTCACGT, si-MTDH-1: AGGAATAAAGGATTCTGAT, si-MTDH-2: AAGTCAAATACCAAGCAAA, and si-MTDH-3: AACTTACAACCGCATCATT.

### 2.12 qRT-PCR

To isolate total RNA from cultured cells and tissues, Trizol reagent (15596026, Invitrogen) was employed. Thereafter, utilizing PrimeScript RT Reagent kit (TaKaRa, RR047A, Otsu, Japan), genomic DNA was removed at 42°C for 2 min, and the resulting RNA sample (1 µg) was converted to cDNA (37°C for 15 min) (reaction volume of 20 µL). Then, qRT-PCR was performed using SYBR Premix Ex Taq II (TaKaRa) on the ABI7500 quantitative PCR instrument (Thermo Fisher Scientific, ABI7500, Waltham, MA, USA). The reaction volume is 20 µL. The primer concentration is 0.5 µM. The cycling program: 95°C for 30 s; 40 cycles of denaturation at 95°C for 5 s, annealing at 60°C for 30 s, and extension at 72°C for 30 s. The number of technical replicates per sample is 3. The comparative 2^−ΔΔCt^ technique was applied to assess target gene expression with GAPDH as the internal control gene. The primer sequences used for quantitative PCR were: MTDH-F: 5^′^-AAATAGCCAGCCTATCAAGACTC-3^′^, MTDH-R: 5^′^-TTCAGACTTGGTCTGTGAAGGAG-3^′^; GAPDH-F: 5^′^-GTCGGTGTGAACGGATTTG-3^′^, GAPDH-R: 5^′^-TCCCATTCTCAGCCTTGAC-3^′^.

### 2.13 Western Blotting Analysis

Total protein from cultured cells were extracted with radioimmunoprecipitation assay lysis buffer (Beyotime, P0013, Shanghai, China) (protein concentration used for loading per lane is 30 µg), tested concentrations using bicinchoninic acid Protein Assay Kit (P0010, Beyotime), and electrophoresed on a 10% sodium dodecyl sulfate polyacrylamide gel, which were shifted onto polyvinylidene fluoride membranes (Millipore, IPVH00010, Burlington, MA, USA). After being blocked in 5% nonfat milk for 2 h at room temperature, the membranes were probed with primary antibodies against MTDH (14065, 1:1000, Cell Signaling Technology, Danvers, MA, USA) and GAPDH (5174, 1:1000, Cell Signaling Technology) at 4°C overnight. Following treatment with matched secondary antibody (7074, 1:1000, Cell Signaling Technology) at room temperature for 1 h the next day, the immunoreactive signals were detected utilizing an imager system (Bio-Rad, ChemiDoc XRS+, Hercules, CA, USA). The grayscale values were analyzed by Image J software (V1.8.0.112, NIH, Madison, WI, USA) after background subtraction. For each sample, MTDH band intensity divided by GAPDH was used to normalize protein expression. The replicate averaging was performed. The number of biological and technical replicates is 3.

### 2.14 Cell Counting Kit-8 (CCK-8) Assay

The cells (1 × 10^3^ cells/well, 100 µL/well) were planted in a 96-well plate in complete RPMI-1640 medium at 37°C. Thereafter, after 48 h incubation, CCK-8 reagent (10 µL, 0037, Beyotime) was introduced into the well for continued culture of 2 h in the dark. Finally, the optical density of each well was determined at the wavelength of 450 nm under a microplate reader (Varioskan™ LUX, Thermo Fisher Scientific) after background subtraction.

### 2.15 Flow Cytometric Analysis

The cells (5 × 10^5^ cells/well) were inoculated in 2 mL of RPMI-1640 medium supplemented with 10% FBS, 100 U/mL penicillin, and 100 μg/mL streptomycin in 6-well plates at 37°C for 24 h. Following various treatments, the cells were harvested, then washed and resuspended in binding buffer. Subsequently, the cells were resuspended in 190 µL of 1 × Annexin V Binding Buffer, followed by the addition of 5 μL AnnexinV-fluorescein isothiocyanate (FITC)/propidium iodide (PI; C1052, Beyotime) using gentle vortexing under dark conditions for a period of 15 min. Finally, at least 1 × 10^4^ cells were collected per sample, and the apoptotic cell percentages were examined using FlowJo software (v10) by flow cytometry (BD Biosciences, FACSCalibur, San Jose, CA, USA).

### 2.16 Colony Formation Assay

To perform the colony formation assay, cells were prepared as a single-cell suspension and seeded into 60-mm culture dishes at densities of 500, 1 × 10^3^, 2 × 10^3^, and 4 × 10^3^ cells per dish. After allowing the cells to adhere for 12 h, the cells were irradiated with doses of 0 Gy, 2 Gy, 4 Gy, and 6 Gy. The irradiated cells were then incubated in a 37°C, 5% CO_2_ incubator for 14 days, with complete medium changes every 3 days. After incubation, the cells were stained with 1% crystal violet (04053158, Solarbio), colonies were observed, and colonies consisting of 50 or more cells were manually counted in each dish.

### 2.17 Immunofluorescence (IF) Staining

The cells were fixed in 4% paraformaldehyde (Maokangbio, MM1504, Shanghai, China) at room temperature for 15 min, then subjected to permeating treatment with 0.3% Triton X-100 (Sigma-Aldrich, 93426, St. Louis, MO, USA) at room temperature for 15 min. After the blockade of 5% goat serum (C0265, Beyotime) at room temperature for 1 h, the cells were exposed to primary antibody against γ-H2AX (ab81299, 1:250, Abcam, Cambridge, UK) overnight at 4°C. Cells were then incubated with secondary antibodies (Goat Anti-Rabbit IgG H&L (Alexa Fluor^®^ 488), ab150077, 1: 1000, Abcam) for 1 h at room temperature in the dark followed by staining of nuclei with 6-diamidino-2-phenylindole (DAPI; 100 ng/mL, D-1306, Thermo Fisher Scientific) for 10 min, with imaging conducted on a Leica fluorescence microscope (Leica, DMI4000B, Wetzlar, Germany).

### 2.18 Statistical Analysis

GraphPad Prism 8.0 (GraphPad Software Inc., V8.0, San Diego, CA, USA) and SPSS 26.0 (IBM Corporation, 26.0, Chicago, IL, USA) were adopted to analyze all *in vitro* experimental data from three independent biological replicates, summarized as the form of mean ± standard deviation. Student’s *t*-test was employed to evaluate statistical significance between two groups, whereas one-way Analysis of Variance (ANOVA) in combination with Tukey’s test was utilized for comparison among multiple groups. Survival was analyzed by the Kaplan-Meier method; predictive value was validated via ROC curves and the AUC. The relation between MTDH expression and clinicopathological parameters in patients with rectal cancer was evaluated using the Chi-square (*χ*^2^) test. A value of *p* < 0.05 was considered statistically significant.

## 3 Results

### 3.1 Feature Selection for Radio-Resistance Related Genes in CRC Using Machine Learning-Based Predictive Modeling

**Figure 1:**
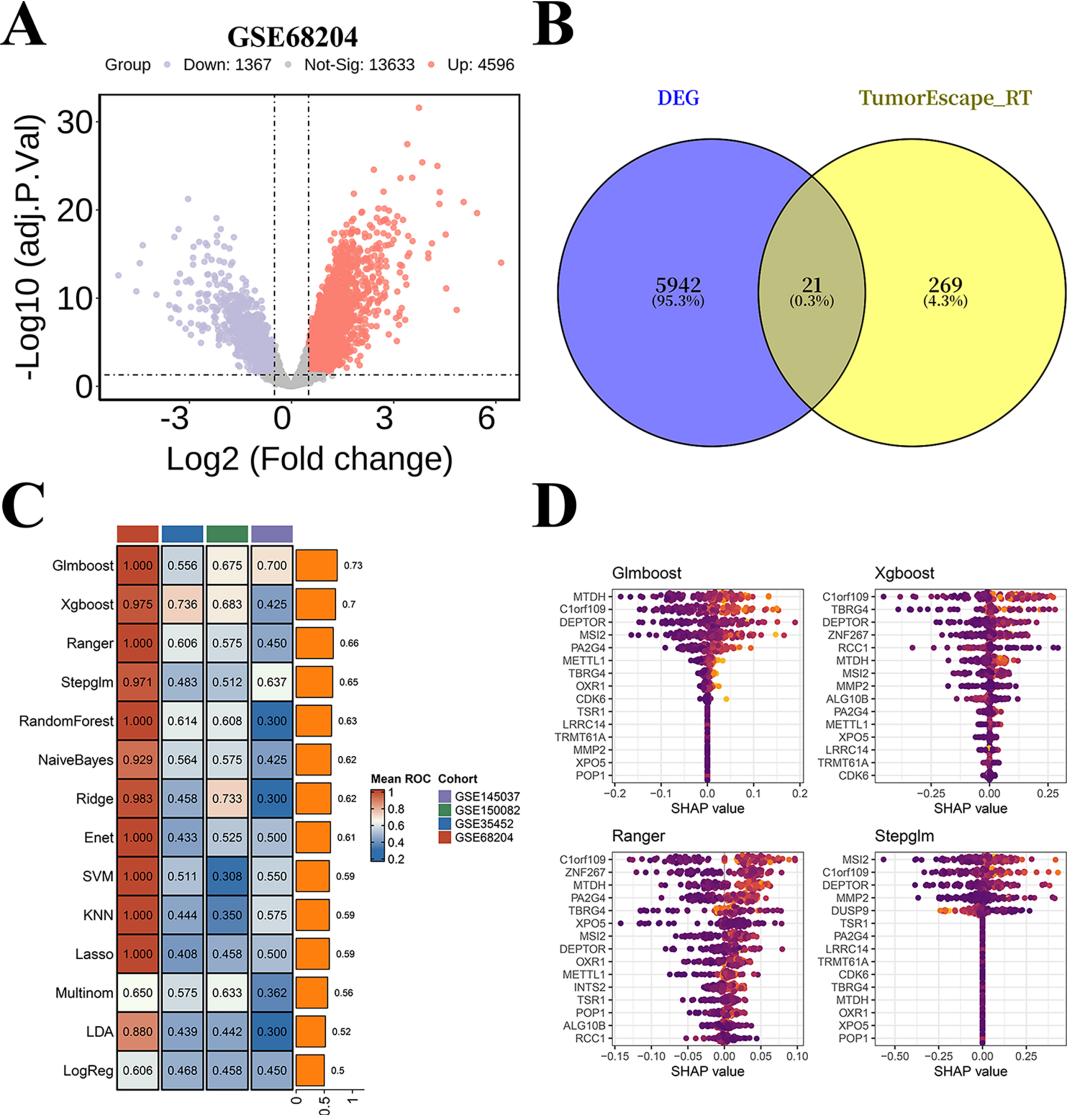
Machine learning-based screening of key genes for colorectal cancer (CRC) radio-resistance. (**A**) Volcano plot of differentially expressed genes (DEGs) between radiosensitive tissues and radioresistant tumors in the GSE68204 dataset. (**B**) The 21 candidate genes were identified via venn diagram. (**C**) A total of 14 machine learning models and the Receiver Operating Characteristic (ROC) of each model across the GSE68204, GSE150082, GSE145037, and GSE35452 datasets. (**D**) Beeswarm plot of SHapley Additive exPlanations (SHAP) values (shows how and how much each gene influences the predictions) in the top-performing machine learning models (GlmBoost, XgBoost, Ranger, and Stepglm) across all cohorts.

In our preliminary analysis to elucidate CRC radioresistance mechanisms, comparative transcriptomics of the GSE68204 cohort revealed 5963 DEGs distinguishing radiosensitive from radioresistant tissues (Fig. 1A). From the MSigDB database, 290 genes associated with radio-resistance were obtained. The intersection of DEGs with known radio-resistance genes yielded 21 overlapping candidates, which proceeded to predictive modeling (Fig. 1B). Using the GSE68204 dataset, we implemented 14 machine learning models and conducted external validation through GEO cohorts (Fig. 1C). SHAP-based summary plots delineated feature importance and directional effects across four optimal models (Fig. 1D). Notably, MTDH consistently demonstrated the highest predictive contribution in all models, establishing it as a pivotal radio-resistance gene in CRC.

### 3.2 Validation of MTDH Expression in CRC Using scRNA-Seq Data

**Figure 2:**
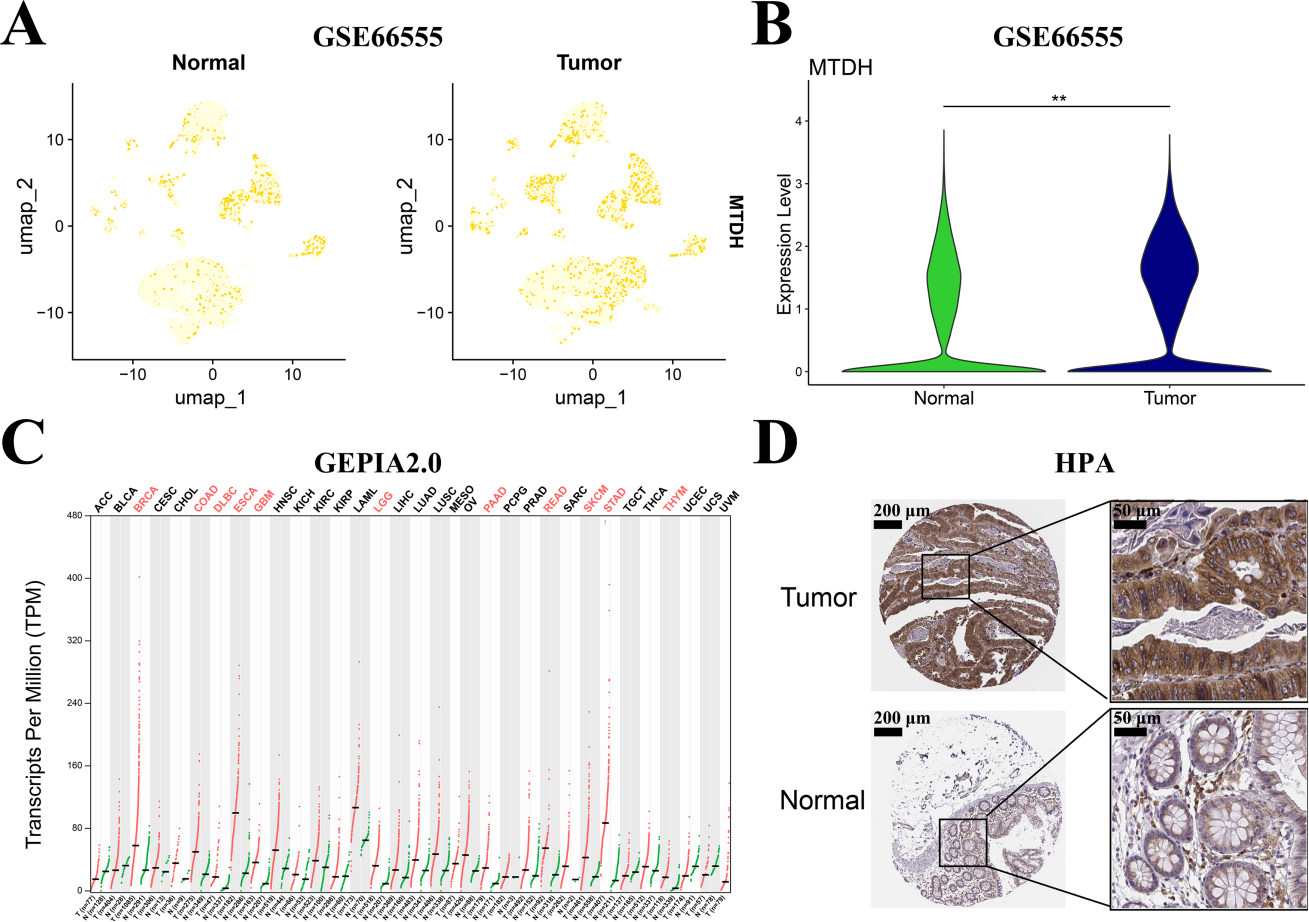
Validation of Metadherin (MTDH) expression in CRC. (**A**,**B**) Uniform Manifold Approximation and Projection (UMAP) visualization and violin plot showing the distribution of MTDH expression between CRC tumor tissues (n = 12) and matched adjacent normal tissues (n = 12) in GSE166555 dataset. (**C**) MTDH expression profiles across tumor samples compared to peritumor samples for 33 The Cancer Genome Atlas (TCGA) tumor types using Gene Expression Profiling Interactive Analysis (GEPIA)2.0. **(D)** Representative Immunohistochemistry (IHC) images of MTDH immunoreactivity in CRC tissue compared to normal tissue via Human Protein Atlas (HPA) database. **p < 0.01.

In the scRNA-seq dataset (GSE166555), samples from 12 CRC patients (including 12 tumor tissues and matched adjacent normal tissues) were analyzed. Dimensionality reduction and clustering of the scRNA-seq data yielded the UMAP visualization shown in Figs. 2A and S1A. Expression analysis revealed significantly higher MTDH levels in tumor tissues compared to normal tissues (*p* < 0.01, Fig. 2B). In addition, the violin plot showed differential MTDH expression in nine cell clusters between tumor tissues and matched adjacent normal tissues (Fig. S1B). Transcriptomic analysis via GEPIA 2.0 confirmed MTDH overexpression in both colon and rectal adenocarcinomas (Fig. 2C). Parallel protein-level validation using HPA immunohistochemistry demonstrated markedly stronger MTDH immunoreactivity in CRC tissues vs. normal counterparts (Fig. 2D).

### 3.3 Validation of MTDH Expression in Diploid Cell Subpopulations Using scRNA-Seq Data

**Figure 3:**
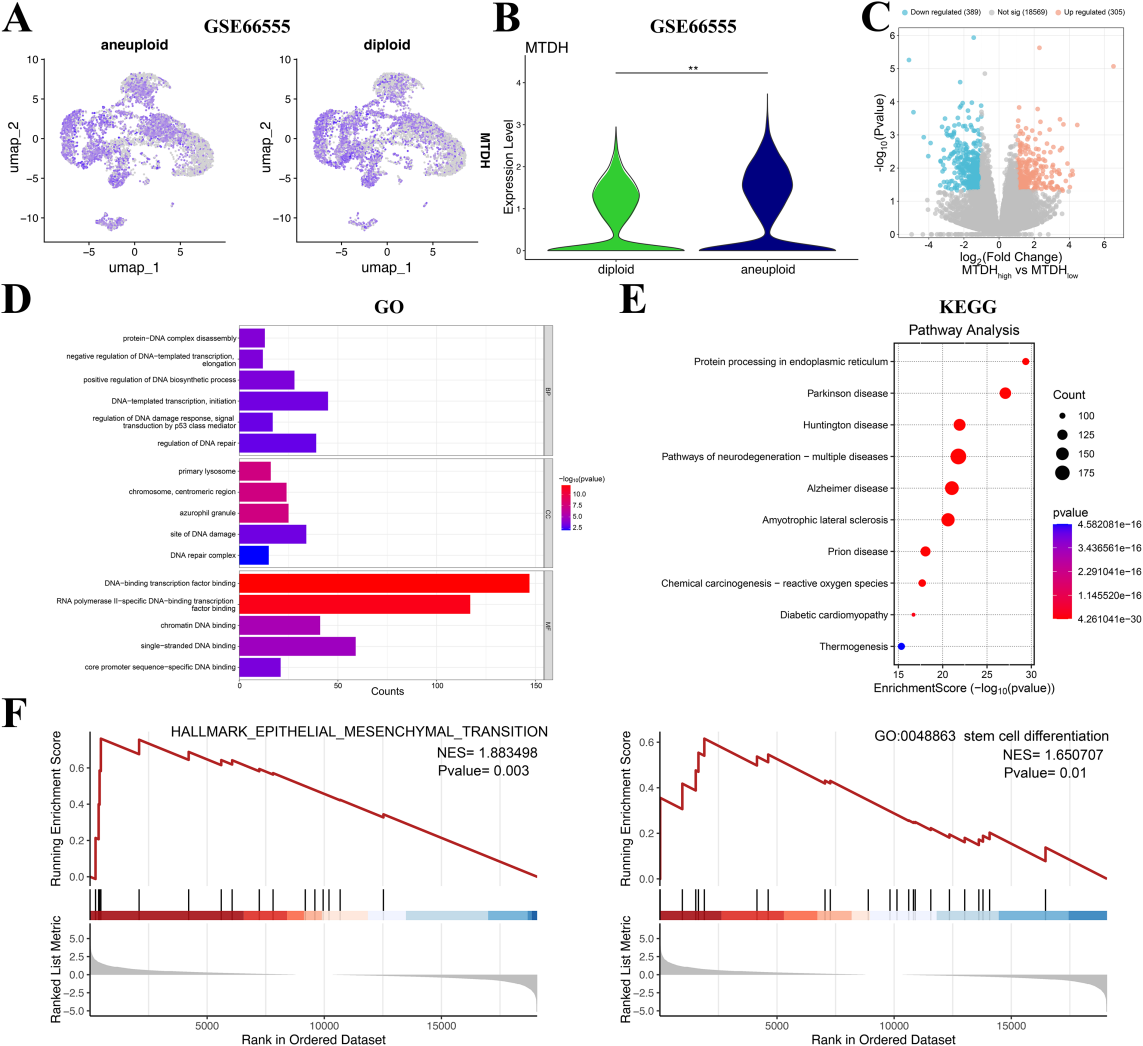
Validation of MTDH expression in aneuploid subpopulation. (**A**,**B**) UMAP visualization and violin plot showing the distribution of MTDH expression between diploid and aneuploid subpopulation in GSE166555 dataset. **(C)** The volcano plot between MTDH high expression and MTDH low expression of aneuploid subpopulation. The screening criterion of *p* < 0.05 and |log_2_FC| > 1. **(D)** Gene Ontology (GO) enrichment analysis of MTDH-related genes. **(E)** Kyoto Encyclopedia of Genes and Genomes (KEGG) Enrichment analysis of MTDH-related genes. **(F)** Enrichment plot of the epithelial mesenchymal transition and stem cell differentiation from gene set enrichment analysis (GSEA). **p < 0.01.

CRC is a malignant tumor of epithelial origin [25]. Normal epithelial cells have antitumor activities [26]. Epithelial cells exhibit apical-basal polarity and cell–cell adhesion. Correct regulation of polarity is essential to inhibit tumor growth [27]. The malignant transformation of epithelial cells in the presence of oncogene activation is generally closely associated with the loss of cell polarity and disorganization [28]. Therefore, it is crucial to distinguish malignant epithelial cells from normal epithelial cells. Currently, an effective approach to distinguish malignant from normal cells involves the identification of aneuploid copy number profiles [29]. Therefore, the CopyKat algorithm was applied to distinguish diploid and aneuploid tumor cell subpopulations of epithelial cells in the single-cell data (Fig. 3A). MTDH expression was significantly elevated in the more malignant aneuploid subpopulation (*p* < 0.01, Fig. 3B). Subsequent analysis identified 694 MTDH-related DEGs between MTDH high expression and MTDH low expression of aneuploid subpopulation (Fig. 3C). Furthermore, enrichment analysis of MTDH-related genes was performed. These genes showed significant GO enrichment in “DNA repair regulation” and “DNA damage response” pathways related to radio-resistance (Fig. 3D). KEGG enrichment results indicated significant involvement in pathways including “Protein processing in endoplasmic reticulum” (Fig. 3E). Also, GSEA analysis showed that MTDH-related genes may be related to epithelial-mesenchymal transition (EMT) and Stem cell differentiation (Fig. 3F). In summary, these findings demonstrate that MTDH was highly expressed in CRC and was associated with radiotherapy resistance in this malignancy.

### 3.4 MTDH Upregulation Is Linked to CRC Radio-Resistance

**Figure 4:**
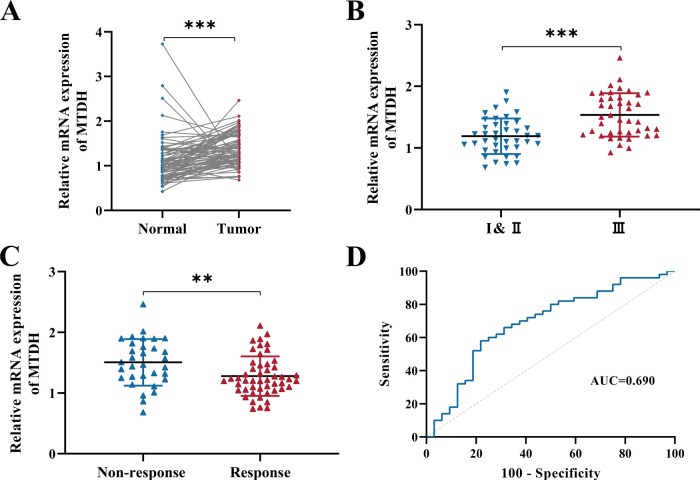
MTDH upregulation is linked to CRC radio-resistance. (**A**) The mRNA level of MTDH in rectal cancer tissues (n = 82) and matched adjacent normal tissues (n = 82). (**B**) quantitative real-time polymerase chain reaction (qRT-PCR) analysis of MTDH expression level in rectal cancer tissues for different Tumor stage (I&II: n = 40, III: n = 42). (**C**) The expression of MTDH in non-response (TRG 2–3, n = 32) and response groups (TRG 0–1, n = 50) was analyzed using qRT-PCR. (**D**) The ROC analysis showed the sensitivity and specificity of MTDH expression to discriminate response (TRG 0–1, n = 50) group and the non-response (TRG 2–3, n = 32) group. **p < 0.01, ***p < 0.001.

We identified MTDH expression in these 82 rectal cancer paired clinical patient samples in order to further examine the clinical characteristics of MTDH in CRC. The comparison results demonstrated that rectal cancer tissues expressed apparently higher levels of MTDH than matched adjacent normal tissues (Fig. 4A). MTDH expression was substantially higher in stage III than in stage I–II disease (Fig. 4B). We then validated the correlation between MTDH expression and TRG, and we discovered that the expression of MTDH was higher in the non-response group (32/82, 39.0%) than that in the response group (50/82, 61.0%) (Fig. 4C), indicating the possible contribution of MTDH to rectal cancer radio-resistance. Table 1 summarizes the general clinicopathological significance of MTDH in rectal cancer tissue expression. Additionally, MTDH abnormal overexpression was remarkably related to tumor differentiation, tumor size, tumor stage, lymph node state, radio-resistance, while it was not closely associated with age and sex (Table 1). Finally, the ROC curve was applied, and the AUC value for MTDH was 0.690 (Fig. 4D, Table 2), suggesting that MTDH is associated with rectal cancer patients’ poor response to radiotherapy.

**Table 1:** Clinical features of the patients with rectal cancer stratified by MTDH expression.

Parameters	Category	n = 82	MTDH Expression		*χ* ^2^	*p*
			Low	High		
**Gender**	Male, n	52	24	28	0.841	0.359
	Female, n	30	17	13		
**Age, years**	≤65, n	46	23	23	<0.001	>0.999
	>65, n	36	18	18		
**Differentiation**	Low, n	39	27	12	11.000	<0.001
	High, n	43	14	29		
**Tumor stage**	Ⅰ-II, n	40	26	14	7.029	0.008
	III, n	42	15	27		
**Tumor size (cm)**	≤5, n	42	31	11	19.520	<0.001
	>5, n	40	10	30		
**Lymph node state**	Positive, n	47	18	29	6.032	0.014
	Negative, n	35	23	12		
**Groups**	Response (TRG 0–1), n	50	31	19	7.380	0.007
	Non-response (TRG 2–3), n	32	10	22		

Note: MTDH, Metadherin.

**Table 2:** AUC results of ROC curve analysis by MTDH in rectal cancer patients.

Group	Index	AUC (95% CI)	*p*	Sensitivity (%)	Specificity (%)	Youden Index
**Non-response group vs. response group**	MTDH	0.690 (0.570 to 0.810)	0.004	58.00	73.13	0.361

Note: AUC, area under the curve; CI, confidence interval.

### 3.5 MTDH is Upregulated in Radioresistant CRC Cells

**Figure 5:**
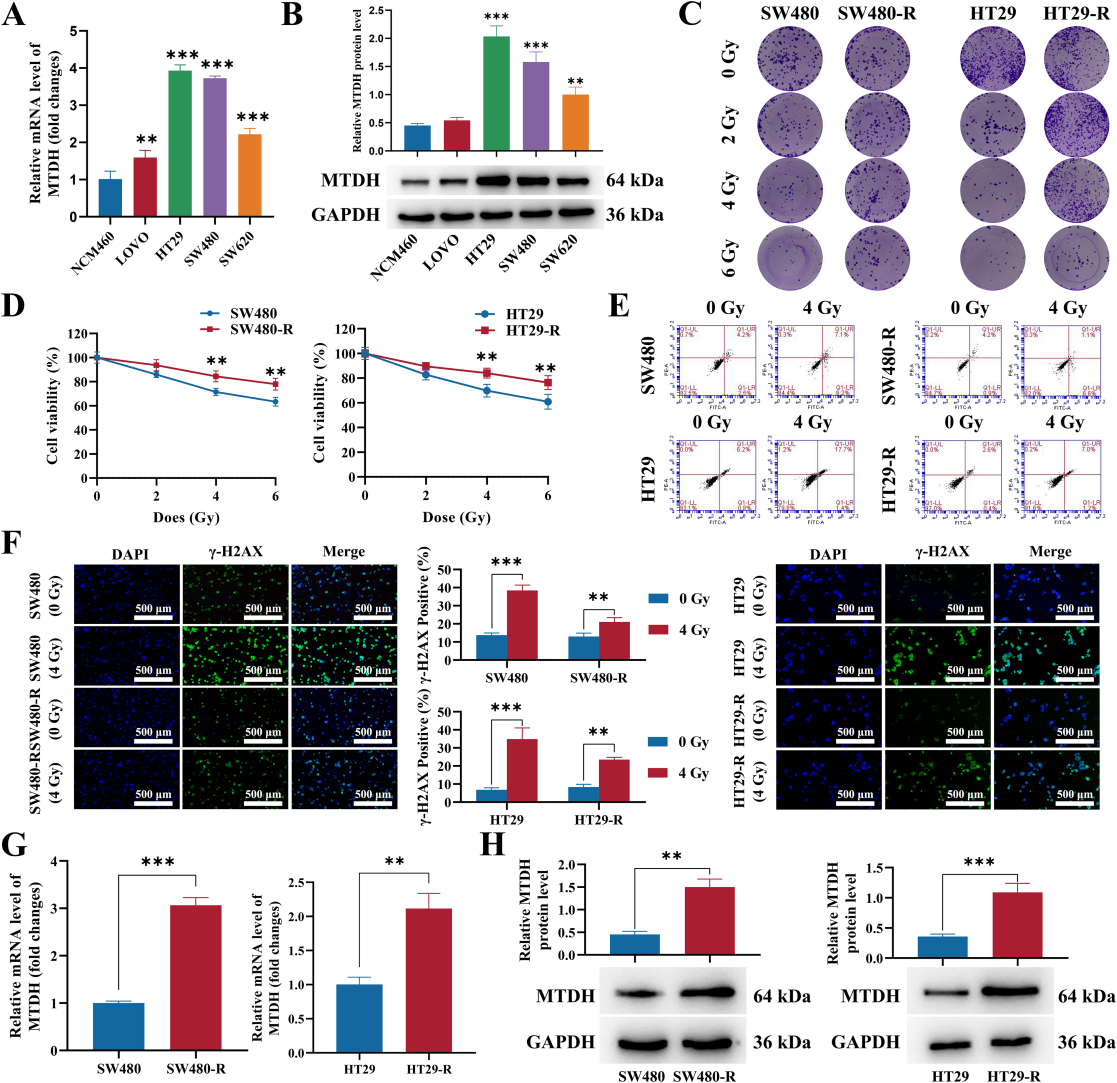
MTDH is upregulated in radioresistant CRC cells. (**A**) The mRNA expression of MTDH in normal cells NCM460 and various CRC cell lines was assessed by qRT-PCR. (**B**) The protein level of MTDH in normal cells NCM460 and various CRC cell lines was determined by western blot. **(C)** Colony formation assays were performed to assess the growth abilities of SW480, SW480-R, HT29, and HT29-R cells. (**D**) The Cell counting kit-8 (CCK-8) assay was utilized to detect the viability in SW480, SW480-R, HT29, and HT29-R cells under 0, 2, 4, and 6 Gy radiation treatment. (**E**) Flow cytometric analysis was carried out to observe cell apoptosis in each group. (**F**) Effect of MTDH on DNA damage validated by immunofluorescence when exposed to radiation (4 Gy). Scale bar represents 200 μm. (**G**) Verifying the mRNA expression of MTDH in radiation-resistant SW480 and HT29 cells and those cells without radiation. (**H**) Verifying the protein expression of MTDH in each group. n = 3/group, **p < 0.01, ***p < 0.001.

Subsequently, we checked the expression levels of MTDH in normal cells NCM460, and CRC cell lines with various degrees of differentiation. Compared to NCM460, both the mRNA and protein expression of MTDH were upregulated in LOVO (undifferentiated), SW620 (poorly differentiated), and particularly in highly differentiated HT29 and SW480 cells showing the highest level (Fig. 5A,B). We next established radioresistant CRC cell lines, SW480-R and HT29-R, followed by evaluation radiosensitivity using CCK-8 assay, flow cytometric analysis, and γ-H2AX staining. Upon irradiation with doses ranging from 2 to 6 Gy, cell viability of SW480, SW480-R, HT29, and HT29-R were decreased in a dose-dependent manner, and two acquired radioresistant cells displayed relatively higher viability and more clone formation than their parental cells (Fig. 5C,D). Moreover, the apoptotic rate was reduced, while the γ-H2AX foci numbers were decreased in both radioresistant SW480 and HT29 cells under 4 Gy radiation compared with normal parental cells (Fig. 5E,F). The above data revealed that SW480-R and HT29-R exhibited greater resistance to radiation than their parental cells. Finally, we examined MTDH expression in radioresistant and corresponding parental cells, and upregulation of MTDH in radiation-resistant SW480 and HT29 cells was found in comparison with those cells without radiation treatment (Fig. 5G,H).

### 3.6 Silencing of MTDH Attenuates the Radiation Resistance of Radioresistant CRC Cell Lines

**Figure 6:**
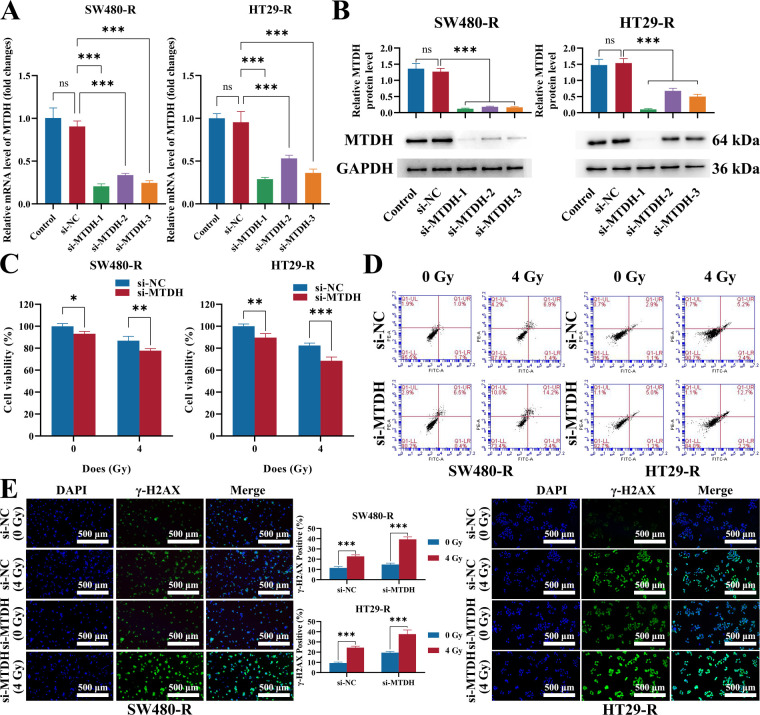
MTDH knockdown alleviates the radio-resistance in SW480-R and HT29-R cells. (**A**) Knockdown efficiency in SW480-R and HT29-R cells was assessed via qRT-PCR. (**B**) Knockdown efficiency in SW480-R and HT29-R cells was verified via western blot. (**C**) The impact of MTDH knockdown on proliferation was validated by CCK-8 assay. (**D**) The flow cytometry assay was conducted to detect the percentage of apoptosis. (**E**) Immunofluorescence was utilized to determine the number of γ-H2AX foci. Scale bar represents 200 μm. n = 3/group, ns = no significant difference, *p < 0.05, **p < 0.01, ***p < 0.001.

In order to further verify whether MTDH modulated the radiosensitivity of CRC, siRNAs targeting MTDH were designed and synthesized in radioresistant cells (SW480-R and HT29-R). The expression level of MTDH was apparently decreased in MTDH-silenced SW480-R and HT29-R cells, with si-MTDH-1 showing the highest silencing efficiency (Fig. 6A,B). Thus, si-MTDH-1 was used for subsequent experiments. MTDH knockdown also significantly impaired the proliferation of radioresistant cells, and proliferative ability was further diminished following irradiation treatment (Fig. 6C). Additionally, radioresistant cells with MTDH silencing exhibited a higher apoptosis rate, particularly further exposure 4 Gy irradiation (Fig. 6D). Meanwhile, γ-H2AX foci numbers were obviously increased in radioresistant cells after MTDH knockdown, while further augmented by radiation treatment (Fig. 6E). Taken together, these results revealed that downregulation of MTDH might alleviate the radio-resistance of CRC cell lines.

## 4 Discussion

CRC remains a heterogeneous disease with an increasing trend in the incidence and fatality by age worldwide [2,30,31]. Radiotherapy is regarded as a crucial method for treating CRC, because radiation not only restrains the development of cancer but also substantially decreases the risk of local recurrence [32]. However, radio-resistance is a major obstacle to hamper the effectiveness of radiotherapy [33]. Therefore, further investigating the mechanisms of abnormally expressed genes underlying CRC radio-resistance is critical to develop novel therapeutic strategies to enhance radiosensitivity. MTDH, as a well-established oncogene, is widely expressed across various cancer types [10]. A high level of MTDH expression in tissues from breast cancer patients can predict a shorter life time [34]. Wang et al. discovered that overexpressed MTDH is observed in liver cancer tissues, which exerts an influence on the prognosis of hepatocellular carcinoma patients [35]. Sultan et al. have found that MTDH is apparently upregulated in advanced tumor grade and stage [36]. However, whether MTDH is related to CRC radio-resistance needs to be studied. This study referred to the research strategies of previously published studies, such as Liu et al., discovering that the voltage-gated sodium channel β3 subunit (SCN3B) is a biomarker for glioma [37].

Consistent with previous reports, our analysis confirmed that high MTDH expression was observed in rectal cancer tissues. In addition, through CRC scRNA-seq data analysis, MTDH expression was significantly elevated in the more malignant aneuploid epithelial subpopulation. GO, KEGG, and GSEA suggested that MTDH-related DEGs associated with “DNA repair regulation”, “Protein processing in endoplasmic reticulum”, “EMT”, “Stem cell differentiation”, and other related pathways were enriched in aneuploid epithelial subsets. Among these, DNA repair is closely related to the metastasis and recurrence of CRC, which is the key factors of CRC progress [38]. Enrichment was observed in “Protein processing in endoplasmic reticulum”, indicating these genes may be involved in immune recognition processes critical for CRC development [39]. EMT is closely associated with tumor invasion and metastasis and has been considered to be a fundamental event in CRC metastasis [40]. Disrupting the balance between stem cell and differentiation programs is a defining property of CRC. As such, genomic alterations that hinder intestinal differentiation, either by activating stem cell-like programs or inactivating pro-differentiation pathways, are central to CRC development [41]. In addition, high MTDH expression was positively linked to several clinicopathological parameters such as tumor differentiation, tumor size, tumor stage, and lymph node status. Further analysis demonstrated that rectal cancer patients with high MTDH expression exhibited shorter survival rates, suggesting that highly expressed MTDH was correlated with an unfavorable prognosis for CRC.

As a membrane protein, MTDH can be targeted by antibodies, which provides the possibility for the clinical development of therapeutic approaches against MTDH [42]. One of MTDH’s most important functional partnerships is with Staphylococcal Nuclease Domain-Containing Protein 1 (SND1) [43]. Several studies have implicated MTDH in promoting tumor growth through association with SND1 [44]. Disrupting this complex compromises tumor initiation in various models, underscoring the potential of the MTDH-SND1 interface as a high-value therapeutic target [44]. A recent study identified a class of small chemical inhibitors that could specifically disrupt the complex. MTDH-SND1 inhibition by these compounds significantly reduces breast cancer progression and metastasis, supporting the therapeutic potential of this new class of inhibitors [45]. Despite this promise, disrupting protein-protein interactions (PPIs) in general, and MTDH–SND1 in particular, poses substantial scientific challenges [46]. Unlike enzyme active sites or receptor-binding pockets, PPI interfaces are often relatively flat, less structured, and feature few deep “hot spots,” making them notoriously difficult to inhibit with traditional small-molecule drugs [47].

A major contribution of MTDH to the carcinogenic process lies in the induction of resistance to standard therapies in a broad spectrum of tumors. For instance, it is found that elevated MTDH expression is adaptive resistance to immunotherapy in patients with hepatocellular carcinoma [48,49]. Higher level of MTDH also contributes to resistance in hepatocellular carcinoma patients who receive sorafenib treatment [50]. Furthermore, the TNF Alpha Induced Protein 2 (TNFAIP2)/MTDH/Twist Family bHLH Transcription Factor 1 (TWIST1) axis holds potential as a therapeutic target for urothelial carcinoma patients receiving cisplatin [51]. In patients with resectable stage III CRC treated with adjuvant chemotherapy, high expression of MTDH may serve as an independent predictor indicating poor outcome [52]. However, the latent role of MTDH in CRC radiosensitivity is still lacking. Our machine learning approach constructed identifying MTDH as a crucial radio-resistance gene in the leading models. Additionally, the expression of MTDH was remarkably higher in radioresistant rectal cancer patients than that in radiosensitive patients, which indicated that higher expression of MTDH was linked to less sensitivity to radiotherapy.

Current cellular research also suggests that MTDH correlates with increased resistance to treatment. MTDH knockdown significantly inhibits breast cancer growth, achieving effective paclitaxel chemotherapy treatment without overt side effects [53]. Furthermore, overexpression of MTDH in A549 cells attenuates the lethal effect of cisplatin, while MTDH deficiency in A549/DDP cells displays the opposite effect [54]. Of note, silencing the expression of MTDH regulates the radiation sensitivity of SKOV3 ovarian cancer cells and reduces their proliferation and metastasis [55]. MTDH loss increases the sensitivity of stem-like medulloblastoma cells to radiation-induced death [56]. AEG-1 (MTDH) deficiency heightened radiosensitivity, diminished proliferation ability, and augmented apoptosis of radiation-treated glioma cells [57]. However, whether MTDH regulates the radiosensitivity of CRC cells remains unknown. In this research, we established radioresistant CRC cell lines, and upregulation of MTDH was found in radiation-resistant SW480 and HT29 cells in comparison with their parental cells. Of note, we demonstrated that downregulation of MTDH could diminish proliferation, augment apoptosis, aggravate DNA damage, and further attenuate the radio-resistance in radioresistant CRC cells. Therefore, these results indicated that MTDH served a pivotal role in radio-resistance in CRC.

However, there are several limitations in this research. Firstly, the identity of aneuploid malignant epithelial subcluster with high MTDH expression could be further identified. Secondly, these results need further validation in a larger and more diverse sample of rectal cancer patients. Thirdly, our present study lacked animal experiments. Finally, we only preliminarily verified the potential role of MTDH for radio-resistance in CRC. Further validation of this conclusion at the protein level by examining the expression regulation of other relevant proteins needs to be provided. In addition, more comprehensive investigations of MTDH related to biological mechanisms involved in regulating tumor radio-resistance and CRC development are required to be elucidated.

## 5 Conclusion

To summarize, our data highlighted that a high level of MTDH in patients exhibited less sensitivity to radiotherapy, and correlated with clinicopathological characteristics and shorter survival rate. Furthermore, MTDH was also upregulated in radiation-resistant CRC cells. MTDH overexpression conferred radio-resistance to CRC cells, while MTDH knockdown partially recovered the sensitivity of radioresistant CRC cells to radiation. Findings of this study suggest that targeting MTDH may provide valuable novel insights into improving the effectiveness of radiotherapy in CRC treatment.

## Supplementary Materials



## Data Availability

The data that support the findings of this study are available from the Corresponding Author, Yesong Guo, upon reasonable request.

## Supplementary Materials

The supplementary material is available online at https://www.techscience.com/doi/10.32604/or.2026.075314/s1.
