# Supplementary material for: Identify MTDH as a Key Gene of Radio-Resistance in Colorectal Cancer Based on Multi-Omics and Experimental Validation
Source: Oncol Res. 2026 Apr 22;34(5):30. doi: 10.32604/or.2026.075314 (PMC13126397; doi:10.32604/or.2026.075314)
Supplement: Supplementary file 1 [file OncolRes-34-75314-s001.docx]

**Supplementary Materials:**


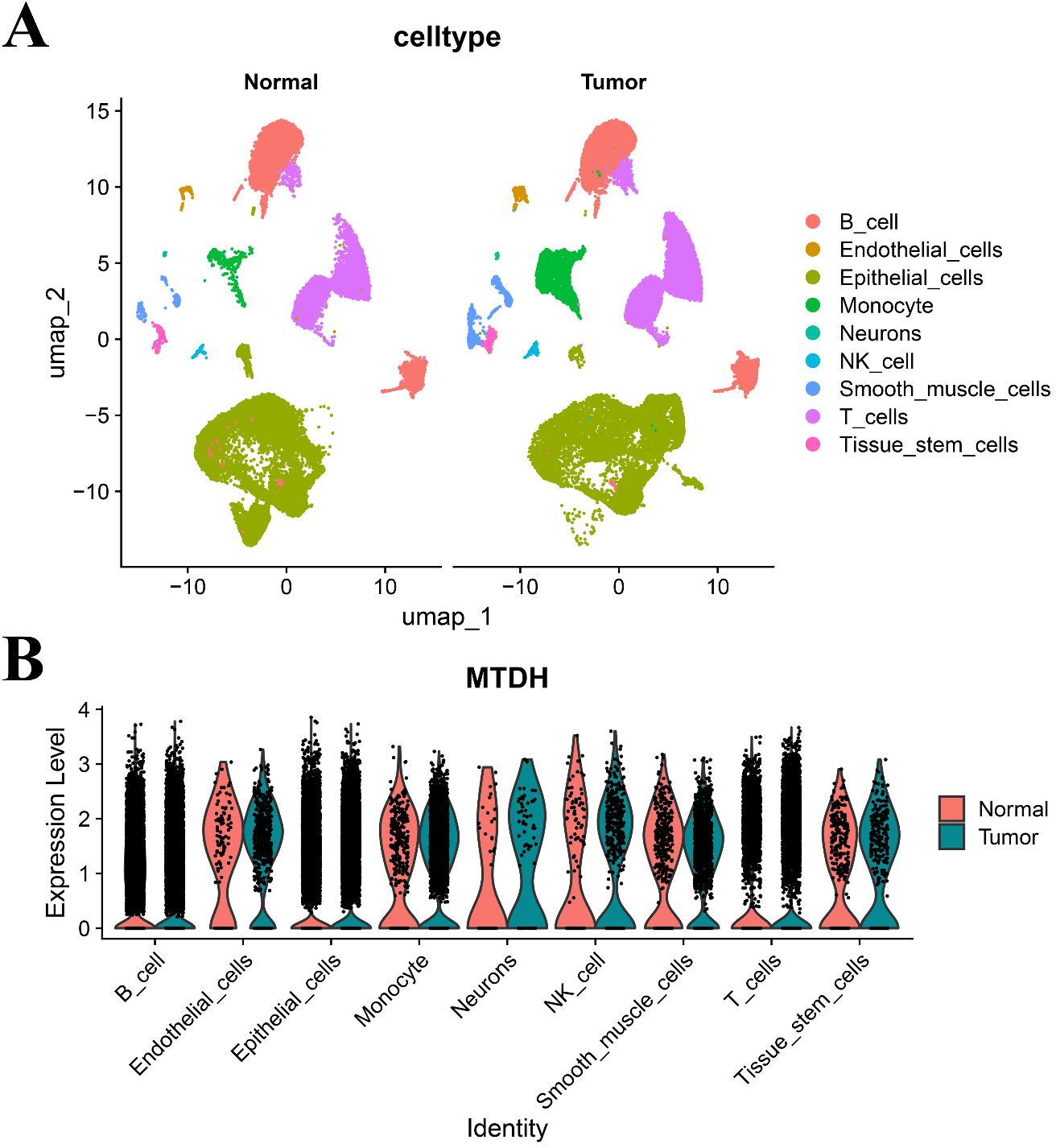


**Figure S1:** (A) A graph represents the distribution of these nine cell clusters across 12 CRC tumor tissues and matched adjacent normal tissues in GSE166555 dataset. (B) Violin plot showing differential MTDH expression in nine cell clusters between tumor tissues and matched adjacent normal tissues.
